# Efficient Sparse Coding in Early Sensory Processing: Lessons from Signal Recovery

**DOI:** 10.1371/journal.pcbi.1002372

**Published:** 2012-03-01

**Authors:** András Lörincz, Zsolt Palotai, Gábor Szirtes

**Affiliations:** 1 Department of Software Technology and Methodology, Eötvös Loránd University, Budapest, Hungary; 2 Sparsense Inc., Boca Raton, Florida, United States of America; 3 ELTE-Soft Ltd, Budapest, Hungary; 4 Center for Integrative Neuroscience, University of Tuebingen, Tuebingen, Germany; Université Paris Descartes, Centre National de la Recherche Scientifique, France

## Abstract

Sensory representations are not only sparse, but often overcomplete: coding units significantly outnumber the input units. For models of neural coding this overcompleteness poses a computational challenge for shaping the signal processing channels as well as for using the large and sparse representations in an efficient way. We argue that higher level overcompleteness becomes computationally tractable by imposing sparsity on synaptic activity and we also show that such *structural* sparsity can be facilitated by statistics based decomposition of the stimuli into typical and atypical parts prior to sparse coding. Typical parts represent large-scale correlations, thus they can be significantly compressed. Atypical parts, on the other hand, represent local features and are the subjects of actual sparse coding. When applied on natural images, our decomposition based sparse coding model can efficiently form overcomplete codes and both center-surround and oriented filters are obtained similar to those observed in the retina and the primary visual cortex, respectively. Therefore we hypothesize that the proposed computational architecture can be seen as a coherent functional model of the first stages of sensory coding in early vision.

## Author Summary

Neural systems favor overcomplete sparse codes in which the number of potential output neurons may exceed the number of input neurons, but only a small subset of neurons become actually active. We argue that efficient use of such large dimensional overcomplete sparse codes requires structural sparsity by controlling the number of active synapses. Motivated by recent results in signal recovery, we introduce a particular signal decomposition as a pre-filtering stage prior to the actual sparse coding, which efficiently supports structural sparsity. In contrast to most models of sensory processing, we hypothesize that the observed transformations may actually realize parallel encoding of the stimuli into representations that describe typical and atypical parts. When trained on natural images, the resulting system can handle large, overcomplete representations and the learned transformations seem compatible with the various receptive fields characteristic to different stages of early vision. In particular, transformations realized by the prefiltering units can be approximated as ‘Difference-of-Gaussians’ filters, similar to the receptive fields of neurons in the retina and the LGN. In addition, sparse coding units have localized and oriented edge filters like the receptive fields of the simple cells in the primary visual cortex, V1.

## Introduction

In the last decades a large body of research has been devoted to explain the nature of neural representations. Since experimental manipulation of the stimuli has the most direct impact on the sensory responses, most of our knowledge comes from studies about the early stages of sensory systems. Although we do not have a complete story yet, experimental and theoretical research did reveal important principles about the nature of neuronal representations together with specific constraints imposed by anatomy and physiology. Derived from the efficient coding theory [1], [2], different popular models – emphasizing redundancy reduction (like [3], [4]) or the sparsity constraint (Sparse Coding, SC, e.g. [5], [6]) – can account for many, but not all relevant features of early sensory processing (e.g. [7], [8]). In this article we argue that a novel computational model of neural representation can be obtained by focusing on one of those relevant features: overcompleteness. For codes with this property the number of potential coding units is larger than that of the input units thus offering increased memory capacity and enhanced robustness against noise and structural perturbations. We will argue that the formation of large and sparse representations of high level of overcompleteness requires adaptive learning which can effectively control the number of active synapses. This structural sparsification has a significant impact on the overall metabolic cost of neural activity. We then present a new sparse coding scheme which is motivated by both theories mentioned above, but is built on a non-conventional signal model assuming an *additive decomposition* of stimuli into “typical” and “atypical” constituents. We also analyze the model's filtering properties when trained on natural images. The main contribution of our study is that principled pre-filtering based on this alternative signal model can indeed facilitate overcomplete SC by supporting structural sparsity. The pre-filtering process is motivated by recent results on efficient compression, completion and decomposition of high dimensional data; computational functions equally important for artificial and natural systems. Based on the finding that our model can simultaneously explain several features of early vision we then suggest a biological implementation of the two stage algorithm.

The paper is organized as follows. In the Results section first we review the computational problem of overcomplete sparse coding and argue about the importance to control synaptic activity. Then we introduce our two stage algorithm which can achieve structural sparsity thus supporting overcomplete sparse coding. In support of our model numerical experiments on natural images are also presented. In the Discussion section we compare the computational properties and biological relevance of our model with alternative approaches. In the Methods section the details of the numerical experiments are provided together with brief descriptions, pseudocodes and references to more elaborate presentations of the algorithmic building blocks.

## Results

In this section we present the problem of (overcomplete) sparse coding (SC) with an emphasis on metabolic constraints (regarding spike activity) and briefly discuss some alternative algorithmic solutions. We then consider if further reduction in computational (metabolic) cost can be accomplished by targeting synaptic activity. Motivated by the insight that the presence of noise hinders the effective control of synaptic activity, we introduce a novel two stage sparse coding algorithm which facilitates structural sparsity (i.e. by keeping the number of active synapses low) and in turn supports the formation of overcomplete sparse codes. The model is then tested on natural images and the responses of the computing units are compared to neural responses in early vision.

### Preliminaries

Due to the high metabolic cost of spiking activity [9]–[11], constraining average spiking rate (over time and population) seems to be a general principle in neural systems (but see [12]). Therefore we also consider sparsity central in our coding model. The objective of the sparse coding (SC) scheme is to find the sparsest representation of the data with low reconstruction error. It has been argued that this scheme offers a computationally and metabolically advantageous trade off between fully localized (like “grandmother”-cells) and distributed codes [13]. Sparse codes essentially try to approximate the underlying hidden structure (the generating sources) of the observed stimulus. The great advantage of SC over other coding schemes is that it directly controls energy consumption by setting the number of active coding units; 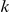 out of 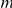 coding units with 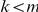 can be active at any given time. Another important property of neural codes is overcompleteness, when the number of coding units (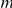) is greater than the number of input units (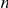, 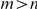). For example, in area 17 of cat the ratio of the output fibers versus the input fibers from the LGN is estimated about 25∶1, while in macaque primary visual cortex, V1 the estimate is between 12∶1 and 160∶1 [14] or even 500∶1 [15]. In principle, overcompleteness provides more flexibility in finding even sparser representations. However, overcompleteness presents a non-trivial challenge for computational models on neural representations. In comparison with biological data, most computational models of SC can find the optimal solution if overcompleteness is 2 to 8-fold at most [16]. Importantly, higher level of overcompleteness may increase the overall metabolic cost of neural coding for two reasons. First, non-optimal solutions require too many iterations thus generating excess spiking activity. Second, overcompleteness induces an asymmetry in the use of the encoder and decoder channels *within* one iteration: while the excitation process requires the use of all 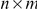 encoder channels, selected subsets of 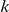 active decoding units require only 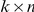 decoder channels. That is methods that avoid the heavy use of encoding are more favorable. The importance of controlling the number of active coding channels (that is the number of synapses which define the receptive field of a neuron) is highlighted by the fact that according to the estimates of [10], more than 50% of the metabolic cost of a single spike can be attributed to the excitatory potentials at the postsynaptic sites (EPSPs). Our goal is thus to find an algorithmic model that can explain overcomplete sparse coding in the brain.

Formally, SC can be stated as an alternating (two step) optimization problem:
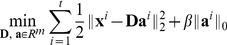
(1)where 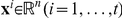 is the 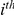 signal, or input to be reconstructed, 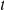 is the number of training inputs, 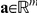 (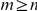) denotes the coefficient vector of the sparse decomposition also called (internal) representation and 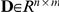 is the basis, or *dictionary* of features. 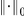 denotes the 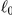-norm, which is the number of nonzero components. The first term minimizes the reconstruction error, while the second one penalizes solutions with many non-zero components. Sparsity of representation 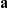 is defined as 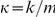 where 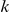 is the number of non-zero components. The resulting code is overcomplete, if 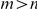 and the difficulty of finding a sparse code with minimal reconstruction error depends on the level of overcompleteness (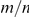) and 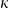. Parameter 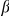 controls the trade-off between the two terms. The reconstruction error or residual may be due to different noise sources that hide the structure of generating sources of the signal.

At one step the basis set is adjusted (*learning process*) to minimize the reconstruction error while the activity of the coding units, 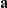 is kept fixed. The straightforward solution would be to let evolve 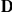 by stochastic gradient on the cost function derived from the reconstruction error, 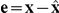 where 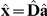 and ‘hat’ denotes the actual estimation. Because of the role of the reconstruction error, this rule is not directly local [17], yet it can be translated [18] into a *set* of Hebbian (local) interactions realized by particular network structures with feedback.

During the selection of non-zero units (formation of the sparse code), features (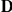) are fixed. However, selection by exhaustive search is a combinatorially hard problem [19]: the number of iterations becomes computationally prohibitive as 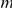 (the dimension of the internal representation) increases. For this reason several approximation method exist, but they either have slow convergence or provide non-optimal solutions. To overcome these limitations, we have chosen a heuristics that combines two approaches. The so called Subspace Pursuit (SP) method [20]–[23] has been chosen because of its superior speed. It is a generalization of matching pursuit [24], which finds local optima in a fast iterative fashion. Importantly, this method is able to discover the global optimum provided that certain conditions are met. Numerical experiments on natural visual stimuli indicate that methods, which assume these conditions, work surprisingly well [25], even though the conditions are unlikely to be met (but see [26] on the inherent limitations of matching pursuit like methods). In contrast to SP, the other algorithmic component – the so called Cross Entropy method (CEM) [27] – is an optimization method designed to find the global optimum. Its main limitation is the slow convergence rate. The combination, termed Subspace Cross-Entropy (SCE) [16] method inherits the best of both worlds: it is reasonably fast and still can yield the optimal solution even at a higher level of overcompleteness. Since we are interested in the formation of sparse codes at very high level of overcompleteness, we used SCE in our numerical experiments. The appendix contains the pseudocodes of SP, CEM and SCE for the sake of reproducibility. Detailed analysis of these methods can be found in [16], [28], [29].

### Improving Overcomplete Sparse Coding

The learning process of Eq. (1) is prune to perturbations: excess activation caused by noise may induce changes in all features thus introducing global (long-range) and low spatial frequency correlations among the features. Such unwanted increase in the number of active synapses implies increased metabolic cost.

Observation noise (e.g. induced by intrinsic neural activity) can significantly decrease the efficiency of OSC as it may easily generate access activation at the output (representation) level, which can only be mitigated by a number of further iterations in order to reduce the reconstruction error. In turn it is essential to counter this effect by actively controlling the number of non-zero components of the filters. This constraint is referred to as *structural sparsity* and implies that visual RFs with *local*, i.e., spatially restricted responses (like the high frequency, concentric RFs of the retinal ganglion cells, the relay neurons in the LGN, or the elongated oriented Gabor patch like RFs of the simple cells in V1) are metabolically more favorable over those that have large global structure with many synapses involved [30]. Approaches like weight thresholding or increasing overcompleteness (see Discussion) fail to address this issue properly. Instead, we turn to an alternative approach by directly separating global (involving many synapses), i.e., *low-frequency* or long-range components of the stimuli *before* the actual sparse coding. Considering the famous 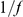 frequency fall of the amplitude spectrum of natural images [31], the low-frequency components carry most of the energy. Principal Component Analysis (PCA, [32], often used decorrelation method), for example, represents the signal in a way that the first component would carry the largest amount of energy, while the last one would carry the least amount. In turn, by applying PCA and then projecting the data *out of the subspace* of the first principal components would yield a representation without the unwanted low-frequency content. Let us remark that this approach is in contrast to conventional thinking which would keep exactly those components with high energy and filter out the rest. While this idea is appealing, PCA based separation of the subspaces strongly depends on the signal statistics: components (“outliers”) with heavy tailed amplitude distribution (characteristic to natural stimuli) can easily break down PCA. In the next section we review a robust alternative to PCA, which can efficiently separate these outliers from the low frequency components. We then propose an overcomplete SC model in which SCE (or any other efficient SC solution) is complemented by this alternative prefiltering as it is expected to support structural sparsity in the subsequent SC stage.

### Two-stage overcomplete SC with structural sparsity

Our concept is based on recent findings of signal processing about recovering low-dimensional data from high dimensional observations [33]. In signal processing, conventional analysis of large dimensional data, such as sensory observations, is often based on the assumption that data have low intrinsic dimensionality: they lie on a low-dimensional subspace. In 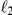 norm (the 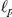-norm of vector 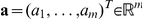, where 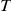 stands for transposition, is defined as 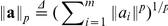), PCA provides rank-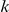 estimate of the data by solving the following problem:
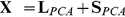
(2)

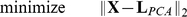
(3)

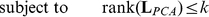
(4)where 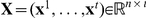 is the matrix of observations (dimension of the observations: 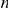, number of data points: 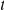), rank of matrix 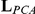 is 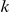 at most and 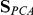 models a small noisy perturbation of each entry 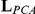. If this perturbation is Gaussian noise, then PCA provides the statistically optimal estimate of the low-frequency, low dimensional subspace 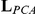. However, deviation from the Gaussian (e.g. gross perturbations or components with heavy tailed distribution) can easily yield incorrect estimates.

Because of the 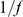 frequency dependence natural stimuli often contain outliers and thus we need an alternative signal model. Let matrix 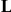 comprise the low frequency components (so it has low-rank as above), while 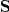 may have full rank, but it is a *sparse* matrix with arbitrarily large entries at random locations: 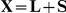. The surprising result is that under certain conditions (on the rank of 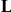 and on the sparsity of 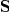) *both* matrices can be *exactly* recovered [33]. Furthermore, it has been proved that efficient recovery is feasible by solving the following optimization problem (Robust Principal Component Analysis, RPCA):
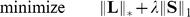
(5)

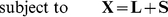
(6)where 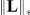 denotes the *sum* of the singular values of 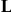, 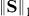 denotes the 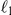 norm of matrix 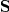, i.e., 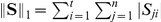. 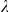 is a trade-off parameter, which governs the dimension of matrix 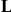. On the other hand, matrix 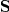 may assume maximal rank, independent of 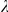.

In addition to robustness against perturbation, the proposed decomposition allows an alternative interpretation of the signals. Instead of treating sparse components as corrupting noise to be filtered, we may consider these outliers as *atypical signals* that carry further information about higher order correlations (like configurational information) not revealed by the low-rank estimate (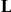). Note that conventional methods (like ICA) would analyze the low rank part only.

The suggested solution (the pseudocode is given in Table 1) iteratively improves the estimation of 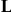 and 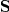 and its computational complexity is only slightly larger than that of the traditional PCA [33]. Another surprising result is that under the assumptions of the theorem, a whole range of 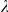 values can return the correct solution, no matter what 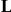 and 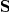 are. A simple reference value for 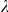 is 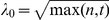 [33] and so we will use a normalized parameter: 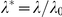.

**Table 1:** RPCA pseudo-code.

initialize:
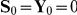 , 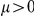
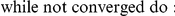
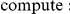
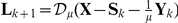
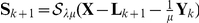
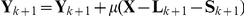
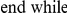
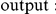 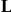 , 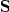 .

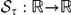
 denotes a shrinkage operator, 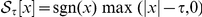 acting on matrices componentwise. For matrix 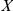, 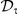 denotes the singular value threshold operator: 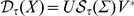, where 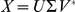 is the singular value decomposition.

Interestingly, as numerical experiments suggest [33], RPCA delivers meaningful signal decomposition even if conditions (about the sparseness of 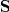) do not hold (like in the case of 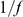 spectra). In these cases, however, different RPCA decompositions can be obtained by setting different 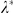 values and 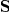 is not guaranteed to be sparse anymore. For this reason matrix 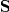 could be the subject of further sparsification. The corresponding sparse coding optimization (see Eq. (1)) in matrix form is given as
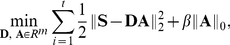
(7)where the matrix 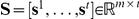 and 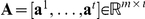, denotes the matrix of the outliers and the matrix of their sparse representations, respectively. The 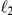 norm based residual may denote full rank observation noise, which implies the following signal model: 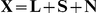. According to [34], it is still possible to give stable estimates for 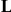 and 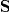, if 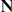 is bounded: 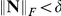, for some 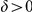 value, where 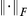 denotes the Froebenius norm. In the demonstrations we opted to use the simpler RPCA model (as in Eq. (6)) without explicit assumptions about the additive noise term.

Let us note that even though the formalism used above is based on matrices, the RPCA procedure can be applied on a single input (thus it may be realized in a neurally plausible form) once an approximation of the low-rank part 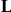 is available. Furthermore, – depending on the input statistics – 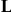 can be approximated even from partial observation by ‘filling in’ missing information [33], [35].

### Computer experiments

To test the impact of RPCA preprocessing on sparse coding, normalized natural image patches were first decomposed by RPCA at different 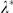 values, then the resulting full rank representations were further encoded by SCE (16-fold overcompleteness with 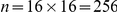 dimensional inputs and 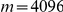 dimensional representation; numerical details are in the Methods Section). We have chosen this particular input set since there already exist a number of computer vision studies on their statistics and the corresponding neural representations under different optimality criteria [14], [31]. The actual overcomplete sparse representations were formed by SCE and the corresponding SC filters were tuned online via stochastic gradient learning. While this level of overcompleteness is still below what has been estimated in the neural sensory systems [15], we believe it is a reasonable choice, as training time is still manageable, yet the results are convincing enough to support the central message of our proposal.

**Figure 1:**
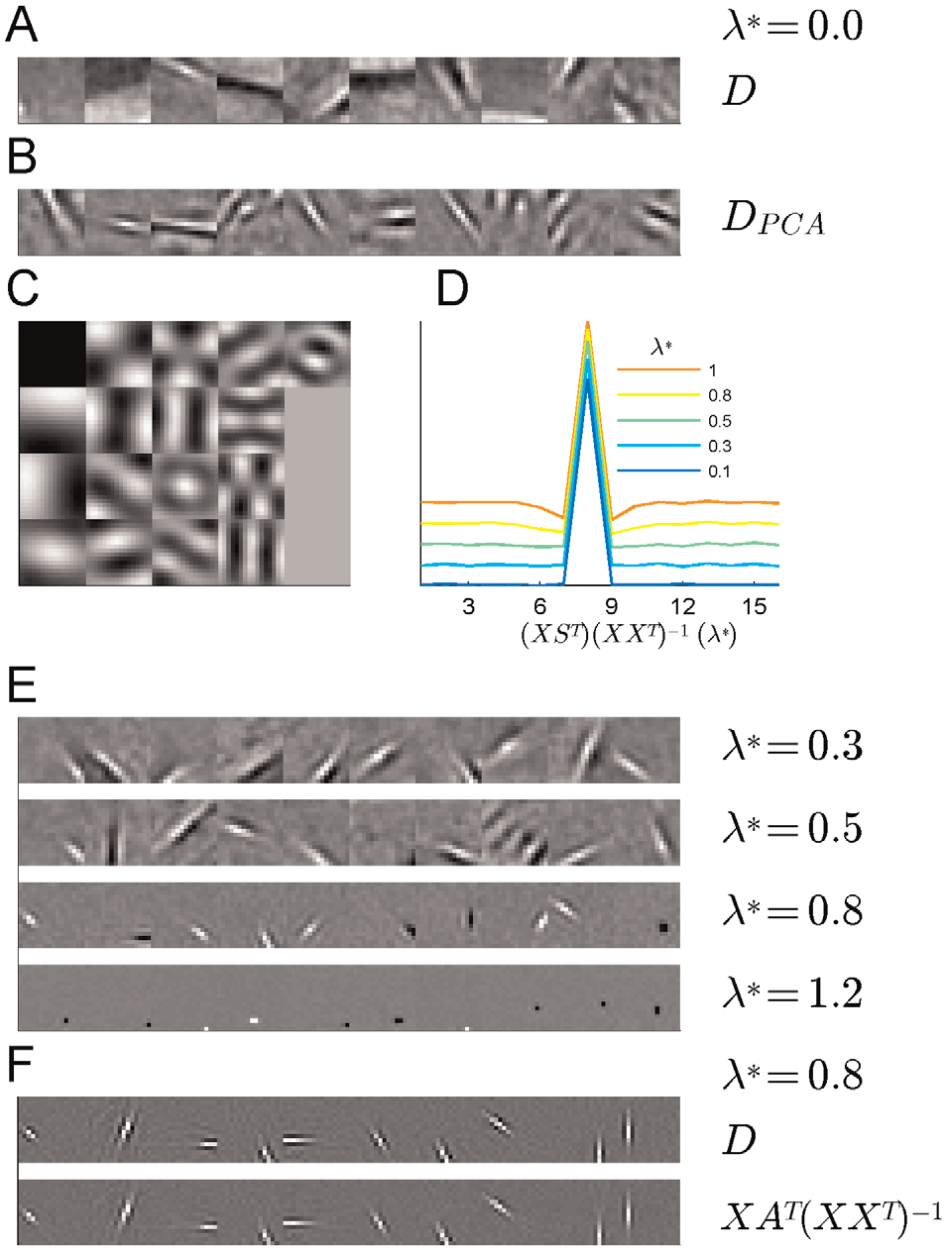
Different basis types of RPCA preprocessing and Sparse Coding. Sample receptive fields are scaled into range [0,1]. (A) no RPCA, columns of dictionary 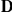. (B) receptive fields learned after PCA pre-filtering: features show wavy, global structure. (C) Features (‘global filters’) of the low dimensional signal for the case 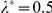 (dimension = 17). (D) reverse correlation of the full rank sparsified signal 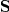 yields stereotypical DoG-like filters with symmetric 2D structure. The figure shows the profile of the central section as a function of 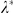. At higher values the negative basin around the peak gets deeper. (E) Randomly selected sparse coding filter sets (over-completeness is 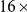, 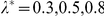 and 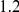) With increasing 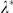 the filters get smaller and more localized (i.e. *cleaner*). (F) For comparison, a set of sparse coding filters (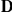) and the corresponding linear approximations (normalized reverse correlation, 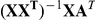) are shown at 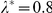.

A few basis features (for sparse coding, 10 out of 4096 columns of matrix 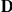) are shown on Figure 1. For visualization purposes each basis vector is scaled into the range 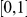 and displayed as a 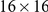 image. Features in the first row of Figure 1A were obtained by conventional SC (applying SCE) without pre-filtering, which corresponds to the case of 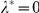.

As we earlier argued, plain SC tends to learn large, global filters, thus preventing the reduction of synaptic cost. Figure 1B plots a few selected SC features when applied on the residuals of traditional PCA. Regarding locality we do not see much improvement: features are still global and manifest large, wavy structures. Figure 1E depicts example filters obtained by applying RPCA prior to SC. Different rows correspond to different 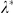 values. The main result of these studies is that the learned basis features get cleaner and more localized, that is, filters get *structurally* sparser as the *single* global parameter increases. On Figure 1F we re-plotted features for 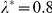 together with the corresponding filters approximated by reverse correlation. Not only the estimation error is smaller compared to the error of the native SC method (Figure 1A), but filters also show larger diversity in their shapes, similar to what has been found experimentally [8]. We also plotted the corresponding filters or RFs of the low-rank signal 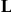 in Figure 1C for 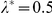, when the number of basis vectors was 17. Figure 1D shows the spatial-dependence of RFs of the sparsified signal 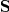 after RPCA for 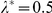.

A surprising result is that the shape of all the obtained RFs for sparsified matrix 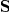 can be described as ‘Difference of Gaussians’ which is the characteristic RF shape [36] of the retinal ganglion cells and the neurons in LGN. The obtained concentric filters 1, are homogeneous and 2, uniformly tile the whole space. Due to their similarity, we show the cross-section of one unit only (Figure 1D). Note that the peaky structure is due to the small image size (discretized DoGs have similar shape at this scale) and more typical DoG shapes could be obtained for larger image patches. We found that for higher 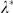 values the negative basin around the peak gets deeper. This development may correspond to the experimentally found developmental changes of the LGN filter profiles in cat [37].

Let us emphasize again that RPCA is not a projection: through an iterative process it extracts the large and sparse components and separates the low-rank part. Interestingly, for natural images, RPCA provides a basis visually almost indistinguishable from those of the PCA filters, but the corresponding representations are different. It implies that PCA may be a good first approximation or initialization for the RPCA iteration method (higher 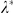 values allow more low-dimensional components).

### Qualitative comparison between filters and RFs

Traditionally, a simple cell RF in V1 is often characterized as a ‘Gabor-patch’ [38]; Gaussian envelope around a cosine wave. To help compare the obtained filters with RFs of real neurons, we also approximated the filters as a Gabor-patch. As 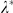 increases the filters become more localized and cleaner, and the Gabor-patch like appearance gets more pronounced. On the other hand, at too large values the filters become small and stereotyped with diminishing harmonic content (see Figure 1E).

**Figure 2:**
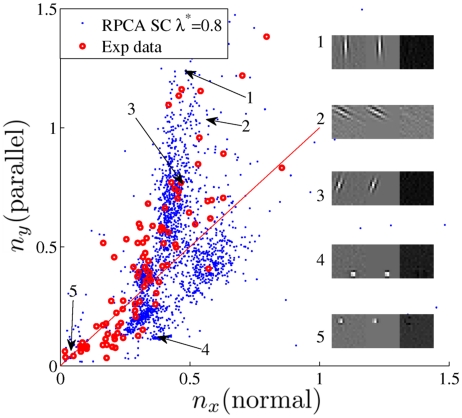
Distribution of the shape parameters for the model and for the experimental data. Receptive fields of simple cells in primary visual cortex, linearly approximated by spike triggered averaging. Data [8] are available at http://web.mac.com/darioringach/lab/Data.html. Our model filters show significant diversity in the fitted shapes similar to what has been found experimentally. While other models (e.g. [39], [40]) are also able to partially match the filters to the observed RFs, a significant difference is that our model uses highly overcomplete representations. For other differences, see the main text.

The distribution of the shape parameters of the Gabor-patch approximations (Eqs. (9)–(11)) is shown in Figure 2 for 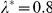. Filters localized at the edges of the 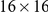 visual space were discarded as their distortion prevents proper fitting. For small filters fitting is imprecise. Filters yielding Gaussian envelope with width less then 0.3 pixel were thus also discarded. It implies that the true number of learned filters at around point 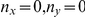 is larger than what is shown in Figure 2. Visual inspection reveals that (i) filters become local and cleaner, (ii) the distribution deviates significantly from the bisection line, and (ii) a considerable portion of the filters is concentrated near the origin 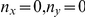. For comparison, we also plotted the distribution of the fitted shape parameters of the experimentally measured RFs of simple cells reproduced from [8]. Considering that we had to drop a number of small filters, the match between numerical and experimental data seems quite good (see, e.g., [39] for comparison), indicating that the proposed model may have biological relevance. Let us note that the observed shape distribution may depend on the level of overcompleteness, but due to the relatively small input size we suspect that further increase in the number of coding units would not result in major changes.

### Numerical analysis of the prefiltering and sparse coding stages

**Figure 3:**
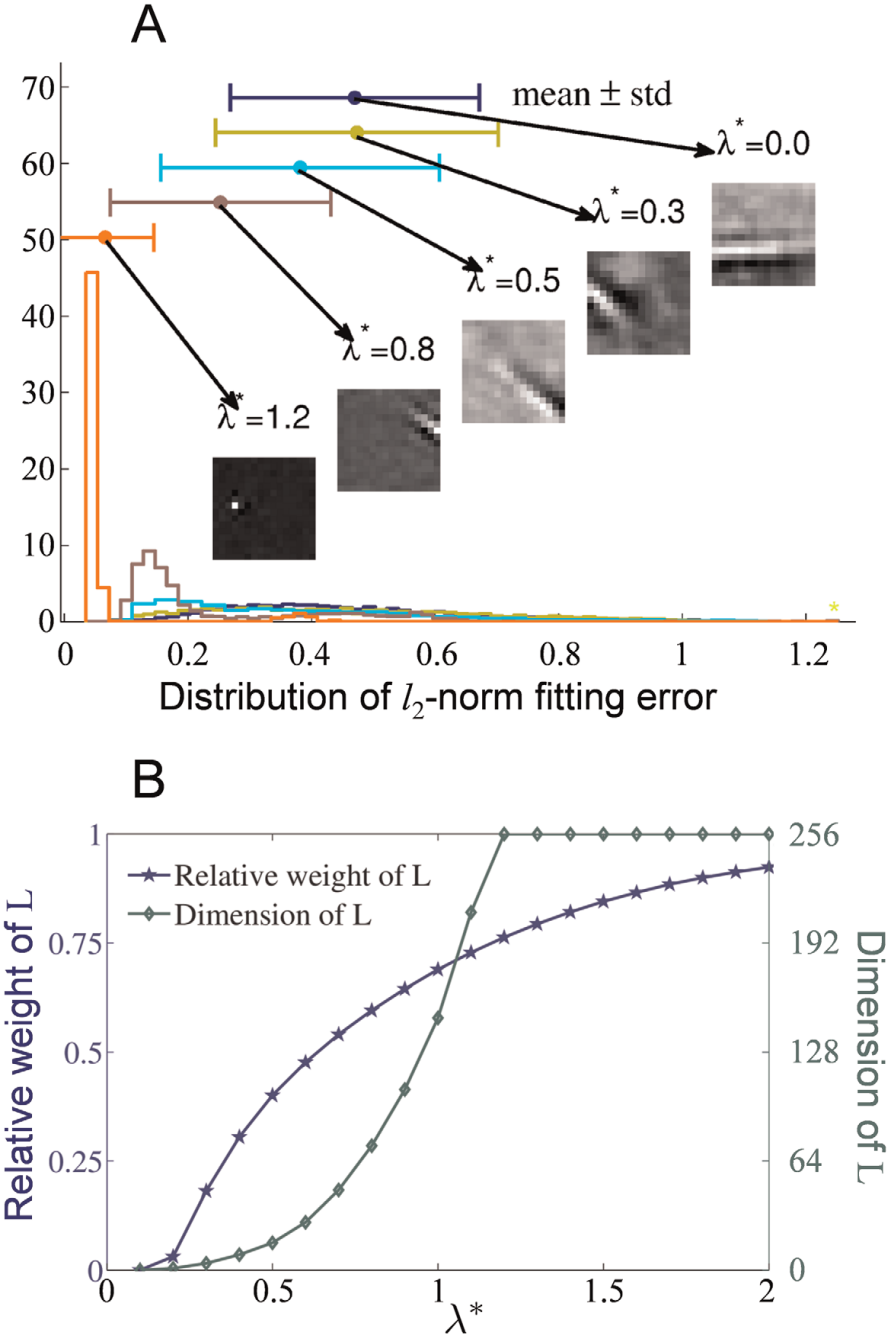
The impact of 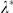 on the signal decomposition and the overall quality of the sparse coding filters. (A) The empirical distribution of the Gabor patch fitting error as a function of 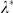. Larger spread signifies deviation from ideal Gabor patch, often used as model shape for experimentally recorded receptive fields. The shift of the mean toward 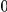 as 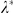 increases is a consequence of the decrease of the average filter size. For each mean value a sample filter is shown demonstrating this shrinkage effect. (B) The dimension and the relative weight of L (the low dimensional signal) in the reconstruction as a function of 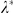. Relevant range is where the dimensionality is low, yet L is able to capture most of the original signal. For image size 16×16 this range is about 0.3–0.8.

Since the assumed signal model is only an approximation for natural image patches, different trade-offs (defined by 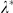 in Eq. (5)) between the contribution of the typical and atypical features to the reconstruction influence the emerging representations after RPCA prefiltering. Figure 3A depicts the influence of 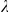 and thus the RPCA decomposition on the statistics of the SC filter shapes as measured by the histogram of the Gabor-patch fitting error. It shows how well the linear approximation of sparse coding filters can be described with a set of oriented Gabor patches often used to characterize experimentally measured receptive fields. If filters have ‘dilated’ global structure then the histogram of the fitting error is probably less peaked. And indeed this is the case: increasing 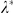 results in more homogeneous, smaller and point-like filters. Let us remark that discretization has a strong contribution to the observed fitting noise.

Figure 3B displays the dependence of the dimension of the low-rank component 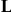 on 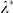 and the relative contribution of 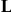 to the reconstruction of the original observations. To calculate the intrinsic dimension of 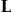, all singular values were zeroed out with amplitude less then 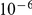 of the maximal amplitude. The important parameter range is where the intrinsic dimension is still low, yet 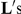 role in the reconstruction is significant. Within that range, 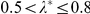 provides the best fit to the experimental data. At higher 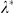 values most of the filters loose their edge-like characteristics.

**Figure 4:**
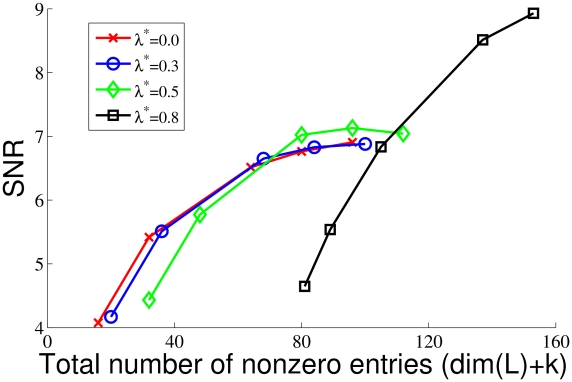
Reconstruction quality as a function of the number of nonzero coding units and 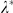. Reconstruction quality is measured by mean SNR: 
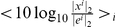
, where 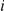 runs over the inputs. Since RPCA is an additive decomposition, the reconstruction error is given as 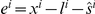. The total number of nonzero entries is given as the sum of the rank estimate of 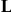 and the preserved number of nonzero units (k) in the sparse overcomplete representation of the atypical part (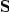) of the RPCA output. Since sparseness level is automatically set by SCE, the following arbitrary values for k were chosen. For 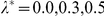 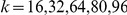 and for 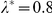, 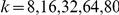.

We have also studied the algorithm's reconstruction ability. Due to the additive decomposition, reconstruction depends on both the “typical” part obtained by RPCA and the overcomplete sparse representation of the “atypical part”. As it is demonstrated on Figure 3 the relative contribution of 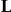 as well as its dimension (number of coding neurons) depends on 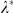. In turn, the fidelity of reconstruction is a function of both the number of units that encode typical features and the number of nonzero entries in the sparse code. Figure 4 displays this dual dependence: reconstruction quality as a function of the total number of nonzero entries, which comprises the rank estimate of 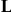 at the given 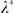 and the preserved number of nonzero entries in the overcomplete sparse representation (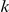). For 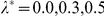 the chosen values were: 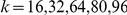 and for 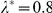, 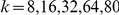. Reconstruction quality is measured by mean SNR: 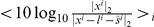. Interestingly, while SNR does not improve much when 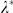 has changed from 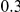 to 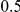, the corresponding filters have significantly changed. Let us note that the overall low values of SNR are due to the fact that no high frequency components have been filtered out prior to decomposition (but see [40], where much higher SNR has been reported after filtering out those high frequency components).

**Figure 5:**
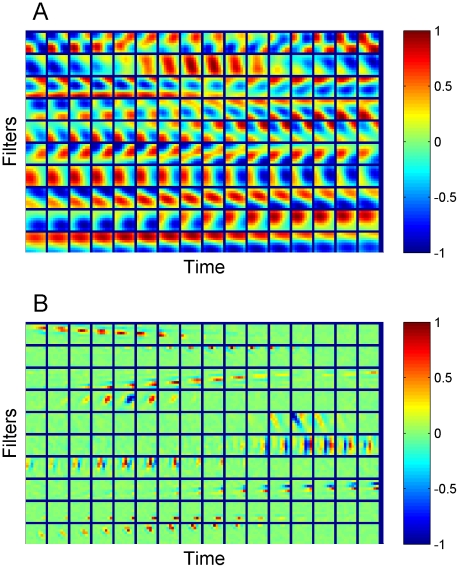
RPCA on concatenated image sequences. Left: The first 10 spatio-temporal filters of the low rank signal,L (rank 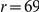) are shown. Each filter is shown as a sequence of 16 frames of size 8×8 pixels. It can be seen that there are spatio-temporally separable as well as non-separable filters. All filters correspond to low frequency temporal or spatial changes Right: 10 selected spatio-temporal filters of the corresponding overcomplete sparse codes that display different spatio-temporal localization and dynamics. While many filters are similar to the presented ones, more training would be needed to achieve similar locality for the majority of filters at this input dimensionality (8×8×16) and level of overcompleteness (16×).

So far we have dealt with static images, but temporal sequences are more realistic: sensory systems are believed to adapt to the spatio-temporal structure of the stimuli. Since RPCA does not rely on prior knowledge about the spatial or temporal arrangement of the data, one expects to see similar decomposition results for data with temporal correlation. For the sake of illustration, temporal correlation was introduced by concatenating 16 image patches of size 8×8 extracted from image sequences on natural scenes. (This was the maximum size we could handle with overcompleteness ratio 16.) Sample filters of the obtained low-rank matrix 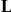 for 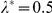 (the corresponding rank estimate is 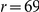) are shown on the left of Figure 5. Filters are ordered by their corresponding eigenvalues. Each filter is composed of 16 frames of size 8×8 pixels. Similar to the filters shown on (Figure 1C), these filters can also be characterized by low spatial and temporal frequency.

The corresponding filters of the atypical parts (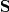, not shown) - as in the static case- are homogeneous, localized in space and time and uniformly tile the visual space. Furthermore, they show Mexican hat like characteristics in the temporal dimension. The regularity may be due to the particular concatenation method we chose.

Sparse coding filters can also be derived from the overcomplete sparse representation of the image sequences after RPCA decomposition. As representations are temporally decorrelated, we obtained filters strongly localized in space and time which resemble to some extent to the receptive field dynamics of simple cells of V1 [41]. A sample set of the obtained sparse coding filters are shown on the right of Figure 5.

It is expected to get better match with experimentally found filters if temporal correlations are introduced into the data model by convolution [42], [43] as opposed to simple concatenation and if nonlinear response properties and nonlinear dynamic interactions are included to handle time warping, for example. These studies go beyond our present goals.

## Discussion

While the resemblance to the biological system is appealing, the original motivation behind applying RPCA was to find means to facilitate the formation of overcomplete sparse representations, an important feature of neural processing that significantly boosts computational efficiency. As we previously argued, *structural* sparsity is needed to control the underlying metabolic cost of the formation of large, overcomplete sparse representations. In principle this control could be realized in different ways. The most straightforward solution would be weight thresholding by zeroing out all filter components (synaptic weights) below an arbitrary threshold value. However, this intuitive regularization may cause more problems than it solves. First, it introduces error for coordinates near zero, e.g. at zero crossing of the response function of a simple cell. In addition, it does not support adaptivity as it may eliminate gradual learning of less frequently represented features. At last it strongly depends on the arbitrary threshold parameter irrespective of the actual input.

Another approach would be to further increase overcompleteness as it might implicitly reduce the number of required components (increased sparsity). However, this idea does not work [44]: when tested on natural images, many filters still show global structure.

We propose RPCA as a particular prefiltering stage prior to the actual sparse coding which indeed facilitates structural sparsity and preserves many useful properties of conventional PCA based decorrelation without its noise sensitivity. Our model may thus resolve the controversy between the hypothesis that PCA like decorrelation should precede subsequent transformations and the fact that the identified RFs cannot be generated by PCA.

Although the proposed RPCA based sparse coding mechanism does not have a biologically feasible implementation yet, its functional relevance may be supported by the following arguments.

The robustness of RPCA has been demonstrated [33] by showing that RPCA yields meaningful representations for different data sets even if the composite signal model cannot be validated (e.g. separation of background (typical) and moving objects (atypical, outstanding features) or separation of face and shadows caused by anisotropic illumination). In particular, for natural stimuli with characteristic ‘scale-free’ statistics (cf. ‘1/frequency’ relation) the conditions of the RPCA theorem are definitely not met as the distinction between low-rank and sparse parts cannot be clearly defined. It may imply that a step-wise incremental separation would be better suited for the input statistics instead of the single layer iterative arrangement of RPCA.

Another important finding is that the RPCA theorem of [33] can be related to recent results on the problem of Exact Matrix Completion [35], which claims that *typical regularities* of a composite signal (represented by columns of 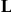) can be completed even from a *small* set of randomly sampled (or partially observed) coordinates of the input. This “sampling advantage” would also improve energy efficiency.

While our model is implicitly supported by the emerging filters, alternative models are also claimed to explain early vision by learning similar features. For this reason we briefly compare a few competing sparse coding models with our proposal.

### Receptive field properties of sparse coding models

**Figure 6:**
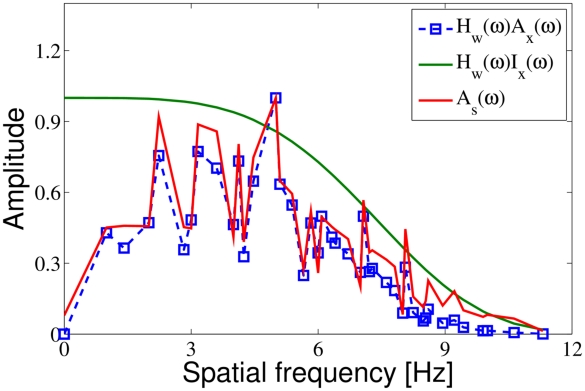
A comparison of the amplitude spectra of the “atypical” output part of RPCA, the whitened input and the whitened ideal input. This plot demonstrates that the particular whitening filter as used in [5], [40] can be seen as a linear approximation of the filtering properties of RPCA when only the atypical output is considered. The thick (red) line is the amplitude spectrum of the RPCA output. The dashed (blue) line with square markers is the amplitude spectrum of the training images filtered with the whitening filter. The thin (green) line serves as a reference: this is the amplitude spectrum of whitened ideal input which has an amplitude spectrum proportional to 1/frequency. Due to the limited input size, there is a natural cutoff at higher frequencies. (Since the size of the images is 16×16, the largest frequency is 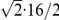.) The whitening filter: 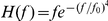, where the cutoff frequency is 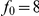. The variances of the plots are due the artifacts caused by the rectangular sampling lattice. For comparison purposes the plots are rescaled onto 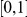.

The biological relevance of neural coding models is often judged by the similarity between their filtering properties and the receptive fields of the corresponding neurons. In the case of visual stimuli, one of the criticisms against theory driven (functional) models (e.g. Independent Component Analysis [4] or Sparse Coding [5]) is the lack of diversity in the filter shapes [8]. This failure might be due to the missing prefiltering stages as seen in the visual pathway. However, nave use of different, biologically motivated prefiltering methods does not seem to offer any improvement, either. For example, applying DoG as high-pass filtering is expected to enhance edge-like features thus yielding a shift of the Gabor-patch shape parameters toward higher values, but the structure of the shape distribution barely changes. Another example is the use of PCA to filter out global features before SC (or ICA), which yields wavy SC basis (Figure 1B). Furthermore, not all filters in V1 have elongated bar shape and most models fail to yield close to concentric shapes found experimentally (for a discussion, see e.g. [39]). As the filter shape distribution on Figure 2 shows, when applied on natural images, RPCA preprocessing *together with* SC delivers the required diversity including the close to concentric shapes. It is worth noting there are other improved coding models (in particular, [40] and [39]) that also claim similarities between the observed and predicted shape distributions of the fitted filters. Our model is similar in spirit to the functional model of [40], whereas the other approach [39] describes a self-organizing system governed by complex dynamics and feedforward inhibition. While the latter one is a promising approach, its dynamics is quite involved and its parameter sensitivity is not known. The other model of [40] is also a sparse coding model and it uses greedy, iterative solutions as mentioned previously. It also uses prefiltering similar to that one used in [5]. They claim the obtained similarity is due to the particular sparsity constraint. For the similar motivations let us remark some differences between the model of [40] and the one proposed here. First, we believe their approach may not be suited to handle large overcompleteness for reasons discussed previously about greedy solutions. Second, the reported difference between the signal to noise ratio of their method and our model is likely due to two factors: we did not employ prefiltering and the overcompleteness in our case is larger. Less sparse codes can encode signals more faithfully then. A fair comparison would be to see the quality of the reconstruction of the high frequency components from sparse codes (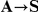), but such comparison would depend on both sparsity and overcompleteness. In turn, an intriguing issue is the optimality of reconstruction quality with respect to the energy consumption. Interestingly, as Figure 6 demonstrates, the linear approximation of the filtering properties of RPCA (seen as the amplitude spectrum of the “atypical” signal part of the RPCA output) looks quite similar to what an ideal whitening filter would yield. This similarity may have the following consequences. First, their result may be attributed both to the particular form of the filter and to the chosen form of sparse coding. Furthermore, it might be the case that such prefiltering behaves as a fast approximation to RPCA. Another difference to mention is that our two-stage model not only provides oriented band pass filters, but it also yields DoG-like filters at the RPCA pre-filtering stage thus providing a simultaneous explanation of two processing stages of early vision. Interestingly, as Figure 6 demonstrates, linear approximation of the filtering properties of RPCA (seen as the amplitude spectrum of the “atypical” signal part of the RPCA output) looks quite similar to what an ideal whitening filter would yield. This similarity may have the following consequences. First, results of [40] may be attributed both to the particular form of the filter *and* to the chosen form of sparse coding. Furthermore, it might be the case that such prefiltering behaves as a fast approximation to RPCA.

### Biological implementation of RPCA based sparse coding

The qualitative agreement between the filtering properties of the early stages of vision and our two-stage algorithm may allow us to attempt to map the algorithm onto the neural substrate by linking the different computational functions to anatomical areas.

An important property of our model is that prefiltering requires a dual representation of the stimuli, which assumption is not in line with the current thinking of hierarchical sensory processing (e.g. [45], [46]), which often comprises alternating filter and pooling operations. So how can we reconcile the assumption on dual representation with single stream models?

Since RPCA implies dynamic interaction between the two emerging representations of the typical (global) and atypical (local) features, decomposition requires either a recurrent network with distinct sub-populations of neurons or two layers with feedforward and feedback connections. As retina does not receive feedback modulations from downstream layers, DoG like filtering of the retinal ganglion cells is not a consequence of RPCA, but it may be explained as a facilitating approximation – as we argued about whitening above – before decomposition. LGN, on the other hand, receives massive amount of feedback from V1. Having learned the filters during early development, it can be assumed that LGN neurons can represent a proxy to the *atypical* features of single stimuli. This representation still contains information about the typical features (since clear decomposition of natural signals is unlikely, due to scale-free statistics). In turn, V1 has a two-fold role in processing. It holds the approximation of the global features extracted from the LGN output and it recodes or re-represents the atypical features in an overcomplete sparse form. A candidate for the first task could be a class of V1 interneurons characterized by large, global receptive fields with weak or no orientation selectivity (e.g. [47], [48]). While it is possible to learn the low-frequency typical parts of new stimulus sets, RFs do not need to be continuously updated as they comprise the most typical correlations of natural images (short term adaptation to quick changes is still required). The second task of overcomplete recoding is then realized by simple cells. This setting thus allows for the alternating substraction of RPCA (Table 1) by the interaction between inhibitory neurons and simple cells in V1 and the neurons in LGN.

In summary, this paper presents a novel two-stage algorithm for efficient overcomplete sparse coding. The proposed robust extraction of low-frequency or typical correlations as a prefiltering step has a few remarkable properties that make the algorithm plausible as an important model of neural information processing. First, it supports the formation of overcomplete sparse codes by effectively controlling the transformation matrices (the synaptic weights) and reducing the number of active synapses. Second, the inclusion of RPCA could significantly facilitate perception as it allows the completion of the typical components even if a part of the stimuli is missing (undersampling, occlusion, cf. exact matrix completion). Since these properties may be beneficial for the nervous system, it would be interesting to see if our algorithm could be realized by biologically plausible neural computations.

## Methods

In this section we briefly present the algorithmic constituents of the subspace cross entropy method used to make overcomplete sparse codes. We also give a short algorithmic description of the RPCA implementation used in the simulations. Finally, details of the training data and the fitting methods are presented.

### OSC Part I: Subspace Pursuit method, (SP)

Subspace Pursuit algorithms have been independently proposed in [23] and [49]. These methods assume that at most 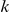 components are sufficient to represent the input. The methods enlarge the subset of candidate features (“candidate subspace”) by 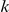 [23] (or 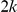 [49]) features and then decrease their number back to 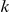 at every iteration. The method of [23] is as follows (the pseudocode is given in Table 2).

**Table 2:** The pseudocode of the Subspace Pursuit method.

input:	
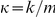 , 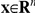	% sparsity and signal
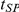	% max iteration number
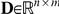	% 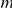 column dictionary
initialization:	
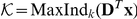	% index set of maximal amplitude elements with set size 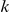
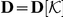	% sub-matrix belonging to index set 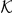
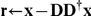	% compute residual
optimization:	
for 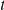 from 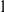 to 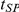	% iteration main loop
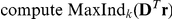	% index set for expansion
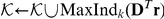	% increase set size to 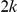 )
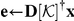	% compute projections
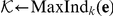	% new index set of size 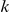
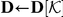	% inserting sub-matrix of index set 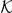
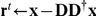	% compute residual
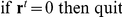	% finish is residual is zero
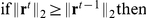	% check for improvement
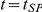	% no new iteration
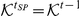	% use previous index set
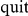	
end loop	
output:	
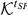	% indices of optimal representation

The goal is to represent the input with minimal reconstruction error using 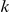 basis only [23]. SP differs from other iterative greedy methods in the incremental refinement of the selected basis subset. First, a representation is generated with the help of the full basis set (using pseudoinverse computations). During iteration 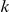 basis are selected based on the amplitude of the corresponding coordinates of the representation. The resulting residual (difference between the original input and the approximation obtained by projecting the representation onto the input space) is then again projected back to the representation space and another set of 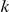 basis are chosen. The two selected subsets are then fused (*expansion*) and the resulting expanded set is used again to project the original input onto the representation space. Finally a new set of 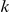 basis are selected by the amplitude of the corresponding coordinates of the projection (*shrinkage*). Iteration stops when the norm of the residual does not decrease anymore. Notation: 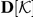 denotes a sub-matrix of 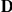 where index set 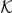 contains the indices of the selected columns. The index set of the first 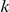 sorted components of a vector 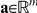 is denoted by 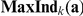.

First, a candidate representation is generated using all basis, then a subset of basis is selected that corresponds to the 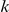 largest components of the representation. This initial selection is then iteratively refined: the residual (that is the difference between the input and its current approximation) is calculated and mapped onto the representation space using the entire basis set again. Then – similar to the initial step – another 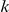 basis are selected based on amplitude of the corresponding components of the mapped residual. The original input is then projected again to the representation space using a 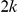 element basis set formed by fusing the two basis subsets. Finally 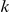 basis vectors are selected again that correspond to the 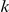 largest components of the projection (basis shrinkage). The iteration stops when the norm of the residual is sufficiently small. SP has superior speed, scaling and reconstruction accuracy over other iterative methods by directly refining the subset of reconstructing (active) components at *each* iteration. Its native shortcomings, though, are the heavy use of the costly encoding transformation of the residuals at each iteration and the preset number of active coding units.

### OSC Part II: Cross-Entropy method, CEM

CEM is a global optimization technique [27] that finds the solution in the following form:
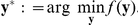
where 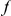 is a general objective function.

While most optimization algorithms maintain a single candidate solution 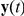 at each time step, CEM maintains a *distribution* over possible solutions. From this distribution, solution candidates are drawn at random. By continuous modification of the sampling distribution, random guess becomes a very efficient optimization method.

One may start by drawing many samples from a fixed distribution 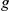 and then selects the best samples as an estimation of the optimum. The efficiency of this random guess depends on the distribution 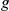 from which the samples are drawn. After drawing a number of samples from distribution 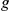, we may not be able to give an acceptable approximation of 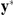, but we may still obtain a *better sampling distribution*. The basic idea of CEM is that it selects the best few samples, and modifies 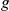 so that it becomes more similar to the empirical distribution of the selected samples. CEM resembles the estimation-of-distribution evolutionary methods (see e.g. [50]) and as a global optimization method, it provably converges to the optimal solution [27], [50].

For many parameterized distribution families, the parameters of the minimum cross-entropy distribution can be computed easily from simple statistics of the elite samples. For sparse representations the Bernoulli distribution is of particular interest [51]. This particular choice may bring about bias towards solutions where sparse components are drawn independently. Derivations as well as a list of other discrete and continuous distributions with simple update rules can be found in [52]. Let us note that we have also translated CEM into an online variant in which parameter tuning is realized by neurally plausible local learning [29]. This translation then allowed us to propose a neurally plausible SC method [28] in which spikes signal the presence of active components, while rate codes encode the corresponding uncertainty of the given component. Since CEM randomly generates candidate sparse solutions hand, it uses a significantly less number of costly encoding transformations. However it updates the probability of all active components similarly, regardless their individual contributions to the actual reconstruction error.

### OSC Part III Subspace Cross-Entropy method, SCE

Subspace Cross-Entropy method (SCE) is an efficient combination of CEM and SP for overcomplete sparse coding. A detailed description can be found in [16] and the pseudocode is given in Table 3. SCE inherits the flexibility and synaptic efficiency of CEM as well as the superior speed and scaling properties of SP without their shortcomings. SCE can be realized by inserting an intermediate control step in CEM to individually update the component probabilities based on their contribution to the reconstruction error. Hence the explicit refinement of the feature set via SP is replaced by an implicit modification through component probabilities.

**Table 3:** Pseudo-code of the subspace cross-entropy (SCE) method for Bernoulli distributions.

required:	
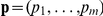	% initial distribution parameters
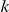	% approximate number of non-zero components
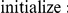 SP 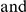 CE	
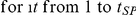	% Main loop of Subspace Pursuit iteration
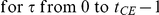 ,	% Main loop of CE iteration
execute CE iteration	
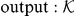	% CE optimized index set
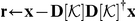	% compute next residual
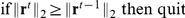	% check for improvement
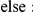	
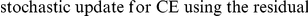	
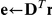	% BU step of Subspace Pursuit
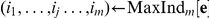	% ordered index set of 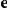
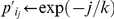	% auxiliary Bernoulli distribution
	with 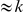 number of 1 s on average
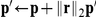	% weigh by residual's norm
	to improve distribution
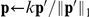	% normalize for 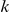 to draw
	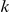 number of 1 s on average
end loop	

For more details, see technical reports [29] and [16].

Since the resulting algorithm is not a greedy method, the algorithm is called as Subspace Cross Entropy (SCE) method without the term ‘Pursuit’.

### Robust Principal Component Analysis

An efficient implementation of RPCA algorithm rephrases the optimization problem of (5) by means of the augmented Lagrangian with the following objective function [33]

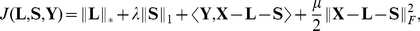
(8)where 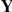 denotes the current residual after subtracting 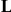 and 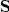. The efficiency stems from the fact that both 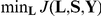 and 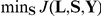 subproblems have simple solutions. Let 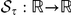 denote 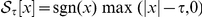, which can be applied componentwise on matrices. For matrices 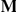, let 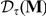 denote the singular value thresholding operator 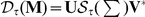, where 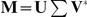 is any singular value decomposition. The corresponding pseudocode is given in Table 1.

### Training data and fitting

The algorithms were trained on 16×16, normalized (zero mean and 1 std) patches extracted from a public database (http://www.cis.hut.fi/projects/ica/data/images/). For the temporal studies, inputs were generated by concatenating 16 normalized patches of size 8×8 extracted from randomly selected parts of publicly available videos (‘football(b)’, ‘garden’, ‘ice’, ‘tempete’, ‘crowd_run’, ‘sunflower’, ‘tractor’; http://media.xiph.org/video/derf/). To speed up calculations, batch learning (50000 samples for static stimuli and 25000 samples for the sequences) was applied to learn the low dimensional subspace of RPCA in the preprocessing stage. On the other hand, to learn the over-complete sparse basis (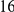-fold over-completeness), 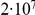 samples have been used. RPCA was run in MATLAB. All other transformations were performed on a cluster of 17 Sony PlayStation 3 consoles in Linux environment using in-house C++ implementation of published algorithms of SVD [53] and CE [27]. The obtained filters were matched with Gabor filters [36], [38] in order to characterize the spatial structures. The Gabor filter parameters are as follows:
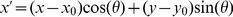
(9)

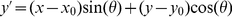
(10)

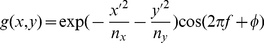
(11)where 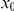 and 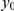 denote the center of the patch, 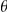 is the orientation of the normal to the parallel stripes of the Gabor function, 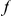 is the frequency and 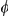 is the phase of the cosine factor, 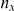 and 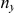 specify the ellipticity of the Gaussian envelope. Fitting was done in MATLAB using the nonlinear least squares optimization function (nsqnonlin(.)) designed for large scale problems. For each parameter value the optimization algorithm was run 20 times with random initialization and the best solution was kept.
